# Bone biochemical markers, bone mineral density, and the risk of osteonecrosis of the femoral head: a Mendelian randomization study

**DOI:** 10.1186/s12891-024-08130-5

**Published:** 2024-12-05

**Authors:** Hai-feng Jia, Ze-ming Tian, Xue-zhen Liang, Han-zheng Li, Bo-wen Lu, Jian Zhang, Gang Li

**Affiliations:** 1 First Clinical Medical College, Shandong University of Traditional Chinese Medicine, Jinan, 250355 Shandong China; 2 Orthopaedic Microsurgery, First College Of Clinical Medicine, Affiliated Hospital of Shandong University of Traditional Chinese Medicine, 16369 Jingshi Road, Jinan, 250014 Shandong China

**Keywords:** 25-hydroxyvitamin D, Alkaline phosphatase, Calcium, Bone mineral density, Two-sample Mendelian randomization analysis, Causal inference

## Abstract

**Background:**

Alterations in bone metabolism may play a significant role in the early stages of femoral head necrosis, yet the causal relationship remains unclear. This study utilizes a two-sample Mendelian randomization (MR) approach to explore the genetic causal links between biochemical markers of bone metabolism, bone mineral density, and the risk of femoral head necrosis.

**Methods:**

This study utilizes publicly available genome-wide association study (GWAS) datasets, with exposure factors including biochemical bone markers (25OHD, calcium, and alkaline phosphatase) and bone mineral density (measured at the lumbar spine, heel, femoral neck, and total body). The outcome of interest is osteonecrosis of the femoral head. We selected validated single nucleotide polymorphisms that are strongly associated with the exposure factors as instrumental variables. Mendelian randomization analysis was conducted using inverse variance weighting(IVW), MR-Egger regression, and weighted median estimation. Additionally, we performed analyses for horizontal pleiotropy, heterogeneity, and sensitivity.

**Results:**

A total of 934 SNPs were included in this study. The MR analysis results indicate that the IVW analysis of 25OHD, Ca, and ALP did not reach statistical significance (25OHD OR = 1.006, 95%CI: 0.69–1.47, *P* = 0.975; Ca OR = 0.856, 95%CI: 0.43–1.70, *P* = 0.657; ALP OR = 1.022, 95%CI: 0.86–1.21, *P* = 0.801). However, bone density, including heel, lumbar spine, and total body bone density, showed a protective causal relationship with the onset of ONFH, while the results for femoral neck bone density did not reach statistical significance (lumbar spine BMD OR = 0.662, 95%CI: 0.48–0.91, *P* = 0.010; heel BMD OR = 0.726, 95%CI: 0.62–0.85, *P* < 0.001; total body BMD OR = 0.726, 95%CI: 0.62–0.85, *P* < 0.001; femoral neck BMD OR = 0.748, 95%CI: 0.53–1.05, *P* = 0.096). Cochran’s Q statistic for IVW and MR-Egger methods indicated no intergenic heterogeneity for all exposure outcomes’ SNPs, and the tests for pleiotropy suggested a low likelihood of pleiotropy in all causal analyses.

**Conclusions:**

The results of this study indicate that there is no genetically mediated causal relationship between serum levels of 25-hydroxyvitamin D, calcium, and alkaline phosphatase and osteonecrosis of the femoral head. However, heel, lumbar spine, and total body bone mineral density can be considered protective factors for the occurrence of ONFH. There is no genetic causality between femoral neck bone mineral density and ONFH development.

## Introduction

Osteonecrosis of the femoral head(ONFH) is a globally challenging ailment, characterized by a high disability rate [1]. It can be categorized into traumatic and non-traumatic femoral head necrosis based on its etiology. Current research [2] suggests that the typical pathological characteristic of ONFH is compromised blood supply to the femoral head, leading to widespread microfractures in the subchondral bone. This ultimately results in subchondral bone collapse, and advanced-stage treatment often relies on hip joint replacement surgery. It is widely acknowledged that high-risk factors for ONFH include systemic steroid use, alcohol abuse, lipid metabolism disorders, radiation therapy, systemic lupus erythematosus, sickle cell disease, and other related conditions [3]. Presently, the number of individuals afflicted with ONFH is steadily increasing, showing a trend toward a younger demographic. It is estimated that in China alone, approximately 8.12 million people suffer from femoral head necrosis [4]. The hip joint pain and impaired mobility caused by ONFH impose a substantial burden on patients, their families, and society at large.

Early detection and effective intervention undoubtedly play a crucial role in preventing the progression of ONFH. Currently, early diagnosis primarily relies on imaging techniques such as X-rays and MRI [5]. Research into the etiology and pathology of ONFH typically focuses on lesions around the hip joint, while there is relatively limited study on whether systemic bone metabolic changes can influence the occurrence and progression of femoral head necrosis.

Bone metabolism generally refers to the metabolic processes of osteoblasts and osteoclasts, which encompass both bone formation and bone resorption [6]. In a normal physiological state, bone formation and bone resorption are in a stable dynamic equilibrium [7]. When this balance is disrupted, the body enters a state of bone turnover disturbance [8]. In recent years, some scholars [9] have proposed a method of monitoring the ratio between bone formation and bone resorption by measuring bone metabolism-related indicators to predict the risk of ONFH. Recognized bone metabolism indicators typically fall into three categories: bone turnover markers, bone metabolism-regulating hormones, and general biochemical markers [10]. These indicators provide a snapshot of the dynamic state of the skeletal system, reflecting ongoing metabolic processes. Since Mendelian randomization studies primarily utilize publicly accessible datasets, biochemical markers, as common laboratory indicators, are widely available and easily obtained. The large-scale data enhances the reliability of MR analysis results. Through a literature review, we have selected the following four indicators as bone-related biomarkers for ONFH: serum 25-Hydroxyvitamin D (25OHD), serum alkaline phosphatase (ALP), serum calcium (Ca), and bone mineral density (including femoral neck, lumbar spine, total body bone mineral density and heel estimated bone mineral density). Current evidence [11] indicates that patients with ONFH have significantly higher levels of alkaline phosphatase compared to healthy individuals (*P* < 0.05). Additionally, alcohol-related ONFH patients exhibit significantly higher levels of 25-hydroxyvitamin D and calcium than those with hormonal or traumatic ONFH. However, the causal relationship between these markers and ONFH remains undetermined.

To mitigate the influence of confounding factors in observational studies, the use of Mendelian randomization (MR) has become increasingly prevalent in assessing causal relationships. MR was first proposed by Katan in 1986 [12], utilizing genetic variations as instrumental variables to evaluate whether a causal relationship exists between exposure factors and outcomes. The fundamental concept of MR analysis is that alleles segregate equally, independently, and randomly during meiosis [13]. Compared to traditional randomized controlled trials, genetic variations are not influenced by confounding factors such as age, gender, or lifestyle. Genome-wide association studies (GWAS) have made significant contributions to the identification of SNPs associated with common diseases, pinpointing multiple genetic loci represented by single-nucleotide polymorphisms. In MR, the exposure factor refers to the variable presumed to affect the outcome, such as biochemical markers or bone mineral density. Single nucleotide polymorphisms (SNPs) associated with the exposure are used as instrumental variables (IVs) to estimate the causal effect, ensuring that the analysis avoids confounding factors. In this study, we employ validated SNPs and aggregated summary statistics from publicly available GWAS datasets, using the MR method to investigate the causal relationships between biochemical markers of bone metabolism, bone density, and the development of ONFH.

## Materials and methods

### Data sources

25OHD, ALP, Ca, and BMD were selected as exposure factors, with ONFH chosen as the outcome. SNPs for each exposure factor were obtained from the IEU Open GWAS database (https://gwas.mrcieu.ac.uk) and the GWAS Catalog database (https://www.ebi.ac.uk/gwas/). The exposure factors are as follows: Ca (Study accession: GCST9001262), 25OHD (GWAS ID: GCST9000061), ALP (Study accession: GCST90025947), Heel BMD (GWAS ID: GCST006979), Total body BMD (GWAS ID: GCST005348), Femoral neck BMD (GWAS ID: ieu-a-980), Lumbar spine BMD (GWAS ID: ieu-a-982). Data related to ONFH are from the Finnish Research Project (https://www.finngen.fi/en), including a total of 1385 ONFH patients and 358,014 control samples. Sample data for exposure and outcome are sourced from European or mixed populations. All data are derived from publicly available GWAS databases, and summary information can be found in Table 1.

**Table 1:** The information of datasets used in our study

Traits	ID	Year	Sample Size	Ancestor	Data sources
Serum 25-hydroxyvitamin D	GCST90000615	2020	417,580	European	GWAS Catalog
Serum Calcium	GCST90012622	2021	62,143	European	GWAS Catalog
Serum Alkaline Phosphatase	GCST90025947	2021	437,896	European	GWAS Catalog
Heel bone mineral density	GCST006979	2019	426,824	European	GWAS Catalog
Lumbar spine bone mineral density	ieu-a-982	2015	28,498	Mixed	IEU open gwas project
Total body bone mineral density	GCST005348	2018	56,284	European	GWAS Catalog
Femoral neck bone mineral density	ieu-a-980	2015	32,735	Mixed	IEU open gwas project
Osteonecrosis of the Femoral Head	finngen_R9_M13_OSTEONECROSIS	2023	359,399	European	Finngen research project

### Selection of the genetic instruments

According to the fundamental principles of Mendelian randomization, three key assumptions must be met: (1) there is a strong correlation between the instrumental variable and the exposure; (2) the instrumental variable is independent of any potential confounding factors related to both the exposure and the outcome; (3) the instrumental variable does not affect the outcome through any pathway other than the exposure. To satisfy these three assumptions, we implemented a series of specific conditions. Initially, all SNPs selected as instrumental variables were statistically significant at the genome-wide significance level (*P* < 5 × 10^− 8^), indicating a meaningful association with the exposure factors. Subsequently, we eliminated SNPs in linkage disequilibrium by setting predetermined parameters (r^2^ = 0.001, kb = 10,000), ensuring the independence of the selected instrumental variables [14]. The SNPs meeting the above conditions were uploaded to the PhenoScanner website (http://www.phenoscanner.medschl.cam.ac.uk/) to exclude pleiotropic SNPs, thereby ensuring conformity with the Mendelian independence and exclusion restrictions. F-statistics were calculated, and SNPs with F-values less than 10 were excluded to prevent bias. Additionally, our choice of publicly available GWAS databases, with their larger sample sizes, helped us mitigate the presence of weak instrumental variables.

### Statistical analysis

We conducted data processing and analysis using the Two-Sample MR package in R software (version 4.3.1). This study employed the Two-Sample MR method for analysis. We employed multiple analytical methods for complementarity to ensure robust results, including the inverse variance weighted (IVW) [15], MR-Egger regression [16], and the Weighted Median (WM) [17]. The causal relationship between bone metabolic indicators and ONFH was assessed using the random-effects IVW model. We also applied WM analysis, which calculates the median of the instrumental variable estimates as a sensitivity analysis. MR-Egger analysis accounts for horizontal pleiotropy and includes an intercept term in the analysis results. Therefore, an evaluation of the intercept term is required to assess bias due to genetic pleiotropy. Effective assessment of instrumental variable assumptions was carried out based on the intercept term analysis results from MR-Egger. We assessed the possibility of horizontal pleiotropy based on the MR-Egger regression results [18], using *p*-values to gauge its presence. If *p* > 0.05, it suggests a lower likelihood of horizontal pleiotropy in the causal analysis, and we conclude that the results are not significantly influenced by horizontal pleiotropy.

Due to differences in analysis platforms, study populations, and SNPs, heterogeneity may exist in Two-Sample MR analysis. To assess heterogeneity, we conducted heterogeneity tests using both the MR-Egger regression and IVW [19], relying on Cochran’s Q statistic and *p*-values for judgment. When *p* > 0.05, it indicates that there is no heterogeneity in the results. Additionally, a leave-one-out sensitivity analysis was performed, whereby each instrumental variable was sequentially excluded, and the MR analysis was repeated using the remaining instruments. This method assesses the impact of excluding each instrumental variable on the causal estimates. The leave-one-out test further confirmed the validity and robustness of the results.

## Results

### Results of instrumental variable selection

Based on the predefined criteria, we obtained instrumental variables that were statistically significant (*P* < 5 × 10^− 8^) for each exposure indicator. We then removed SNPs in linkage disequilibrium (r^2^ < 0.001) and extracted SNPs that corresponded to ONFH from the GWAS database. Subsequently, we uploaded the obtained SNPs to the PhenoScanner website to eliminate confounding factors. These SNPs were finally included in the MR analysis, resulting in a total of 934 SNPs. Among them, there were 85 for 25OHD, 9 for Ca, 322 for ALP, and 518 for BMD (15 for FN-BMD, 420 for HEEL-BMD, 16 for LS-BMD, and 67 for TB-BMD).

### Results of two-sample MR analysis

**Fig. 1:**
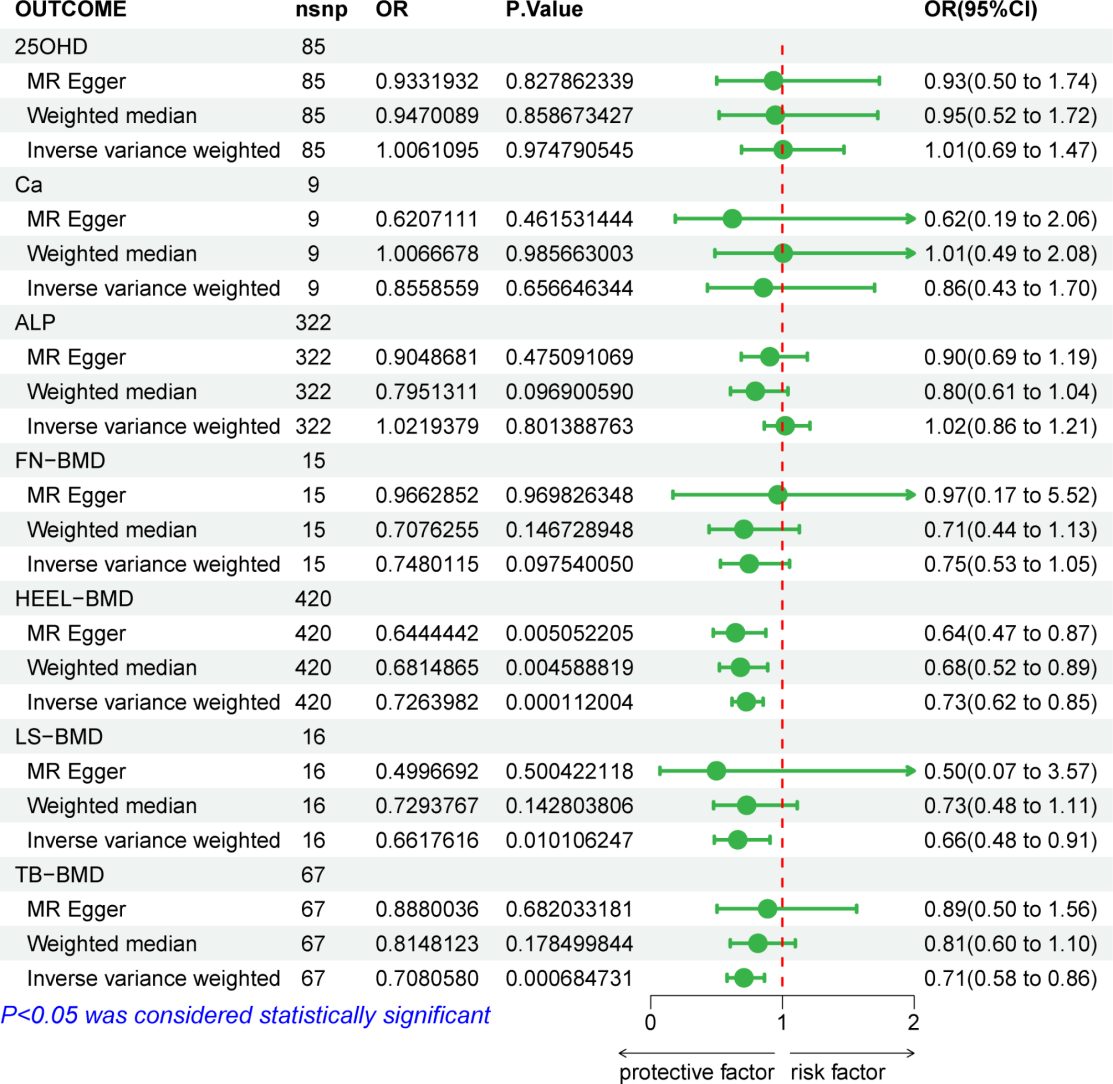
Mendelian randomization results: bone biochemical markers, bone mineral density, and osteonecrosis of the femoral head

MR analysis was primarily conducted using the IVW, MR-Egger, and WM methods to assess causal relationships. IVW was considered the final judgment method, with the other two methods providing supplementary reliability to the results. The analysis results were mainly interpreted based on OR values and *p*-values. When OR > 1, we considered the exposure factor as a risk factor for the disease, and conversely, when OR < 1, the exposure factor was considered a protective factor. Results with *p*-values less than 0.05 were deemed to indicate a significant causal relationship. All three analysis methods consistently showed that 25OHD (OR = 1.006, 95%CI: 0.69–1.47, *P* = 0.975), Ca (OR = 0.856, 95%CI: 0.43–1.70, *P* = 0.657), and ALP(OR = 1.022, 95%CI: 0.86–1.21, *P* = 0.801) are not causally related to ONFH, as their *p*-values were all > 0.05. The MR analysis of bone mineral density suggested that Heel BMD (OR = 0.726, 95% CI: 0.62–0.85, *p* < 0.001), lumbar spine BMD (OR = 0.662, 95% CI: 0.48–0.91, *p* = 0.010), and total body BMD (OR = 0.708, 95% CI: 0.58–0.86, *p* < 0.001) can all be considered protective factors for ONFH. In other words, higher bone mineral density values are associated with a lower likelihood of ONFH, indicating a causal relationship between exposure and outcome. However, femoral neck BMD was not found to have a causal relationship with femoral head necrosis (OR = 0.748, 95%CI: 0.53–1.05, *P* = 0.096), and summary information can be found in Fig. 1.

### Result of heterogeneity and horizontal pleiotropy test

The Cochran’s Q test indicated that there was no heterogeneity in the analysis results for 25OHD, Ca, ALP, and BMD (*p* > 0.05). The pleiotropy test also showed that the results did not exhibit horizontal pleiotropy (*p* > 0.05). Detailed test results can be found in Table 2. Furthermore, we conducted a leave-one-out validation, where the MR results remained stable with minimal changes after sequentially excluding individual SNPs, demonstrating the robustness of the results.

**Table 2:** The result of sensitivity analyses of MR

Expose	IVW estimates		MR-egger pleiotropy test	
	Cochran	*p* value	MR-egger intercept	*p* value
Serum 25-hydroxyvitamin D	76.45	0.708	0.002	0.766
Serum Calcium	10.88	0.209	0.025	0.535
Serum Alkaline Phosphatase	328.86	0.369	0.005	0.269
Heel bone mineral density	426.34	0.392	0.004	0.365
Lumbar spine bone mineral density	7.36	0.947	0.019	0.781
Total body bone mineral density	65.73	0.486	-0.013	0.405
Femoral neck bone mineral density	9.20	0.818	-0.016	0.774

## Discussion

In this study, we utilized the Two-Sample MR analysis method and public databases from large-scale GWAS studies to investigate the causal relationships between bone biochemical markers, bone mineral density, and osteonecrosis of the femoral head. The results demonstrate that there is no genetic causality between the levels of 25OHD, Ca, ALP, and ONFH. Among the bone mineral density indicators we selected, four of them showed positive causal relationships with ONFH, except for femoral neck BMD.

In the early stages of femoral head necrosis, particularly in ARCO stage I or earlier, some patients often manifest only localized pain, soreness, and other discomfort around the hip joint. This is attributed to the recognition of necrotic bone and damaged tissue by the host immune system as foreign entities, such as type I collagen and non-collagenous matrix proteins, thereby eliciting an immune response that aims to facilitate bone repair [4, 20]. At this stage, magnetic resonance imaging (MRI) may not yet reveal significant alterations. It has been reported [21] that in the early stages of ONFH, alterations in bone mineral-matrix occur, characterized by a decrease in phosphate to amide ratio and carbonate to amide ratio compared to normal populations, along with a localized reduction in bone mineral density. Research [22] has shown that abnormal proliferation and differentiation of bone marrow mesenchymal stem cells can lead to an imbalance in bone metabolism, resulting in the destruction of trabecular bone structure, which may be a significant factor in the development of ONFH. It has been reported that relevant indicators of bone metabolism may be valuable in the detection and diagnosis of femoral head necrosis [9, 23].

25OHD, Ca, and ALP are closely associated with bone metabolism and are widely utilized as biochemical markers in clinical practice. 25-Hydroxyvitamin D represents the active form of vitamin D and plays a dual role in bone metabolism. It can stimulate mature bone cells to engage in bone remodeling while simultaneously inhibiting the differentiation of immature bone cells [24]. This results in increased activity of osteoblasts and inhibition of osteoclast function, ultimately promoting bone growth. Some studies [25] suggest that 25OHD plays a positive role in local bone reconstruction within the femoral head and contributes to the deposition of bone minerals such as calcium and phosphorus. Previous studies [26] have reported a negative correlation between the levels of 25-hydroxyvitamin D and the risk of ONFH, with 25OHD deficiency being considered a risk factor for ONFH.

Calcium serves as the fundamental building material for bone synthesis in the human body, with 99% of calcium stored in the bones and teeth [27]. It has been reported that in the pathological examination of patients with alcohol-induced femoral head necrosis, the lesions primarily exhibit localized bone sclerosis and necrosis [28]. Therefore, in the early stages of alcohol-induced femoral head necrosis, calcium levels may experience an increase. In an experimental model of hormone-induced femoral head necrosis using New Zealand white rabbits, the model group exhibited significantly lower calcium levels compared to the control group [29].

Serum alkaline phosphatase (ALP) is primarily derived from bone and the liver and is widely utilized in the clinical screening of various diseases. Bone alkaline phosphatase (B-ALP) plays a critical role in biomineralization and is considered a biological marker of bone formation. ALP, present in osteoblasts, supplies phosphate for the deposition of hydroxyapatite and can reduce pyrophosphate levels, thereby promoting bone growth [30]. Consequently, when liver function is normal, ALP can reflect the activity of osteoblasts. In a retrospective study [11] involving 401 ONFH patients and 81 healthy subjects, ONFH patients exhibited higher serum alkaline phosphatase levels than healthy individuals. This increase was believed to occur during the repair process following femoral head necrosis. However, the results of this study establish that the levels of 25OHD, Ca, and ALP are not causally related to ONFH.

In recent years, there has been a growing body of research on the correlation between femoral head necrosis and bone mineral density, with the majority of studies suggesting a negative association between femoral head necrosis and bone mineral density. Our study also confirms this. In our research, we selected lumbar spine, heel, femoral neck, and total body bone mineral density for MR analysis. The results demonstrate that, except for femoral neck bone mineral density, the other three indicators all exhibit a protective causal relationship with femoral head necrosis. In other words, higher bone mineral density in these respective areas is associated with a lower likelihood of developing femoral head necrosis.

It is worth considering that the femoral neck, being the closest region to the femoral head, may increase the risk of hip fractures significantly due to reduced bone mineral density, which can subsequently lead to secondary femoral head necrosis. However, in the context of our research results, it does not appear to function as a protective factor.

In a prospective study [31] involving 243 non-traumatic ONFH patients and 399 healthy subjects, it was found that ONFH patients had significantly lower bone mineral density in the lumbar spine and femoral neck compared to healthy individuals. This aligns with our research findings regarding lumbar spine bone mineral density. However, in our perspective, there is no genetic causality between femoral neck bone mineral density and the occurrence of ONFH. Undoubtedly, a decrease in femoral neck bone mineral density significantly increases the risk of femoral neck fractures. The compromised blood supply resulting from femoral neck fractures undoubtedly adds to the risk of femoral head necrosis. Nevertheless, based on our results, a reduction in femoral neck bone mineral density does not directly impact the occurrence of femoral head necrosis. Similarly, Lin’s research [32] reported that there was no significant difference in femoral neck bone mineral density between the collapse and non-collapse periods of femoral head necrosis.

Our research results indicate that when bone mineral density decreases, femoral head necrosis may occur correspondingly. Therefore, we have reason to believe that in the early stages of femoral head necrosis, the occurrence of femoral head necrosis can be detected through bone mineral density testing. To our knowledge, our study is the first MR study on the genetic causal effects of bone metabolism markers, bone mineral density, and the risk of ONFH. Two-sample MR studies have several advantages, as they can reduce the interference of confounding factors and reverse causality that are often present in previous observational studies. Furthermore, by rigorously selecting instrumental variables strongly associated with the exposure factor and excluding SNPs related to confounding factors, we can best fulfill the assumptions of Mendelian randomization.

This study employed MR to evaluate the causal relationship between exposure and outcome using genetic variants as instrumental variables. It is important to note that MR findings predominantly reflect causal relationships at the genetic level, specifically the effect of genetically predicted changes in the exposure on the outcome. These results may not directly translate to the effects of modifying the exposure through environmental, behavioral, or therapeutic interventions, which are influenced by non-genetic factors. Therefore, while MR provides robust evidence for causal inference by minimizing confounding and reverse causation, further research using complementary study designs, such as randomized controlled trials or long-term cohort studies, is necessary. These studies could explore whether modifying the exposure through postnatal interventions (e.g., lifestyle changes, pharmacological treatments) yields similar effects on the outcome. Such integrative approaches would offer a more comprehensive understanding of the exposure’s modifiability and its implications for disease prevention and management.

## Limitations

However, our study also has some limitations. (1) The GWAS datasets we collected did not differentiate by gender, which could potentially introduce gender-specific biases into the results. Additionally, the majority of the study participants included in the research were of European descent, so the study outcomes may have some biases when applied to other racial or ethnic groups. (2) There are still large gaps in GWAS data for certain biomarkers or the genetic basis of diseases. This lack of data limits our possibilities to perform Mendelian randomization analyses in certain directions. For example, B-ALP is a more specific marker of bone metabolism; however, due to the limited availability of genetic data related to B-ALP, we had to rely on SNPs associated with total ALP for our causal analysis. Although total ALP is not entirely equivalent to B-ALP, it can still provide insights into bone metabolism to a certain extent.(3) Considering the limited number of SNPs available for femoral neck bone mineral density (FN-BMD) (*n* = 15), we acknowledge the possibility that the lack of a significant association may represent a Type 2 error (false-negative). The confidence interval (CI) for FN-BMD largely fell below 1, suggesting that a small causal effect may exist but could not be detected in this study due to insufficient statistical power. Researchers should dig deeper into the potential relationship between femoral neck bone mineral density and ONFH and make reasonable hypotheses in the future. Future studies with a larger number of SNPs or more comprehensive GWAS data may be better equipped to detect such an effect and clarify the potential causal relationship with ONFH.

## Conclusion

In conclusion, this study has shed new light on the causal relationship between biochemical markers of bone metabolism, bone mineral density, and ONFH through MR analysis. Specifically, this study has yielded the following conclusions: serum 25OHD, serum Ca, and serum ALP levels do not exhibit a genetic causal effect on ONFH risk. Regarding bone mineral density, lumbar spine BMD, heel BMD, and total body BMD demonstrate a protective causal relationship with ONFH, meaning that higher bone mineral density is associated with a reduced risk of ONFH occurrence. There is no genetic causal relationship between femoral neck BMD and the occurrence of ONFH.

## Data Availability

Publicly available datasets were analyzed in this study. These datasets can be found at the following URLs: IEU OpenGWAS database (https://gwas.mrcieu.ac.uk/); GWAS Catalog database (https://www.ebi.ac.uk/gwas/);FINNGEN Research Project (https://www.finngen.fi/en) .
